# IFN-γ licenses normal and pathogenic ALPK1/TIFA pathway in human monocytes

**DOI:** 10.1016/j.isci.2024.111563

**Published:** 2024-12-10

**Authors:** Amandine Martin, Solène Caron, Mélissa Marcotte, Pauline Bronnec, Etienne Garneret, Nora Martel, Georgina Maalouf, Pascal Sève, David Saadoun, Yvan Jamilloux, Thomas Henry

**Affiliations:** 1 CIRI, Centre International de Recherche en Infectiologie, Inserm U1111, Université Claude Bernard Lyon 1, CNRS, UMR5308, ENS de Lyon, University Lyon, F-69007 Lyon, France; 2 CeRéMAIA: Centre National de Références Maladies Autoinflammatoires et Amylose Inflammatoire, 69000 Lyon, France; 3 Internal Medicine, University Hospital Croix-Rousse, Hospices Civils de Lyon, 69000 Lyon, France; 4 Department of Internal Medicine and Clinical Immunology, Sorbonne Universités, AP-HP, Groupe Hospitalier Pitié-Salpêtrière, Centre National de Références Maladies Auto-immunes et Systémiques Rares, INSERM, UMR S959, Immunology-Immunopathology-Immunotherapy (I3), 83 Boulevard de L’hôpital, 75013 Paris, France

**Keywords:** Natural sciences, Biological sciences, Immunology

## Abstract

Alpha-kinase 1 (ALPK1) is an immune receptor sensing the bacterial nucleotide sugar ADP-heptose. ALPK1 phosphorylates TIFA leading to its oligomerization and downstream NF-κB activation. Specific mutations in *ALPK1* are associated with an autoinflammatory syndrome termed ROSAH and with spiradenoma (skin cancers with sweat gland differentiation). This study investigated ALPK1 responses in human mononuclear cells and demonstrates that human mononuclear cells have distinct abilities to respond to ADP-heptose. Notably, IFN-γ is required to license the ALPK1/TIFA pathway in monocytes, while it was dispensable for the responsiveness of B cells. IFN-γ induced *TIFA* upregulation in monocytes, and *TIFA* induction was sufficient to recapitulate the licensing effect of IFN-γ. IFN-γ treatment promoted the phenotypic expression of pathogenic *ALPK1* mutations. The licensing effect of IFN-γ in monocytes was blocked by JAK inhibitors. These findings underscore the critical role of IFN-γ in ALPK1 function and suggest JAK inhibitors as potential therapies for ALPK1-related inflammatory conditions.

## Highlights

• ADP-heptose (ADP-H) responsiveness differs between different human blood cell types
• ADP-heptose responsiveness in monocytes is dependent on IFNγ priming
• TIFA is induced by IFNγ in monocytes. TIFA induction is sufficient for ADP-H responsiveness
• JAK inhibitors block TIFA induction and may inhibit pathogenic ALPK1 activation in ROSAH

## Graphical abstract

**Figure:**
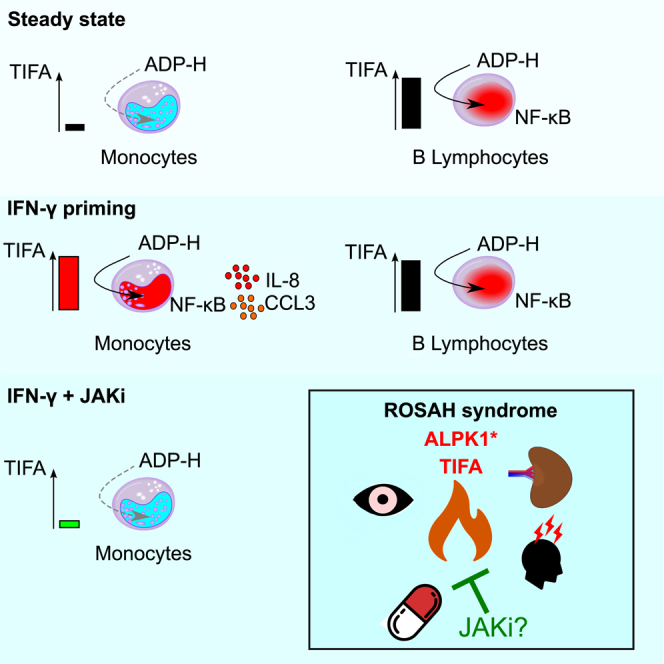

## Introduction

Alpha-kinase 1 (ALPK1) is an innate immune receptor that senses ADP-heptose, a bacterial metabolite associated with the lipopolysaccharide (LPS) biosynthetic pathway.^1^^,^^2^ Upon ADP-heptose binding, ALPK1 phosphorylates TRAF-interacting protein with forkhead-associated domain (TIFA) at threonine 9, leading to the oligomerization of TIFA and downstream activation of TRAF6 and the canonical IκB kinase (IKK) complex.^3^ IKKβ phosphorylates IκBα, leading its degradation and to the release of NF-κB. Additionally, direct phosphorylation of NF-κB p65 serine residues 536 and 529 controls the kinetics of its nuclear translocation^4^ and enhances its transcriptional activity,^5^ respectively. Active nuclear NF-κB induces the transcription of numerous pro-inflammatory genes, including IL-8, CCL3, and TNF.

ALPK1/TIFA axis is activated upon infections with several Gram-negative bacteria, including *Shigella flexneri*,^2^ *Helicobacter pylori*,^6^ and *Yersinia pseudotuberculosis*.^1^ ALPK1 promotes lung inflammation and bacterial clearance during *Burkholderia cenocepacia* infection.^1^ In addition, the commensal bacterium *Akkermansia muciniphila* activates the ALPK1/TIFA pathway in the gut, contributing to the maintenance of the epithelial barrier.^7^

Mutations in *ALPK1* can also lead to a monogenic multisystem disease termed ROSAH syndrome. ROSAH stands for retinal dystrophy, optical nerve edema, splenomegaly, anhidrosis, and migraine headache.^8^ ROSAH syndrome has been recently recognized as an autoinflammatory syndrome.^9^^,^^10^^,^^11^^,^^12^ However, it is not known whether the damage observed in the various organs is due to the ALPK1/TIFA/NF-κB pathway or to a moonlighting function of ALPK1. ROSAH syndrome belongs to the spectrum of relopathies (i.e., diseases impacting NF-κB activity) but differs from most of these diseases^13^ suggesting that the specific symptoms observed may be due to a specific expression pattern of the ALPK1/TIFA pathway or to a specific regulation of this pathway.

A recurrent mutation, p.V1092A, in *ALPK1* has also been identified in spiradenoma and spiradenocarcinoma, two forms of skin cancers with sweat gland differentiation.^14^ Overexpression of the two variants associated with ROSAH syndrome (p.T237M and p.Y254C) and of the variant associated with spiradenoma (p.V1092A) trigger NF-κB overactivation.^12^^,^^14^^,^^15^ Other *ALPK1* mutations were identified in patients suffering from periodic fever with aphthous stomatitis, pharyngitis and cervical adenitis (PFAPA),^16^ another autoinflammatory syndrome. The relevance of these mutations in disease remains to be determined. Interestingly, the ROSAH and spiradenoma ALPK1 protein variants phosphorylate TIFA in response to endogenous (i.e., self) nucleotide sugars (e.g., UDP-mannose) while “wild-type” (WT) ALPK1 does not.^15^ This observation suggests that these mutations trigger NF-κB activation and autoinflammation by promoting recognition of self sugars, while WT ALPK1 recognizes only the nonself sugar, ADP-heptose, originating from bacterial metabolism.

The knowledge on ALPK1 signaling in human blood cells is currently very limited,^17^^,^^18^ despite the key potential role of monocytes in triggering systemic autoinflammatory syndromes. Here, we investigated ALPK1 signaling in peripheral blood mononuclear cells (PBMCs) to decipher the regulation of the pathway in different cell types, understand the underlying mechanisms, and assess the therapeutic potential of drugs targeting these mechanisms for ROSAH syndrome’s patients.

## Results

### IFN-γ licenses ALPK1/TIFA pathway in monocytes

**Figure 1:**
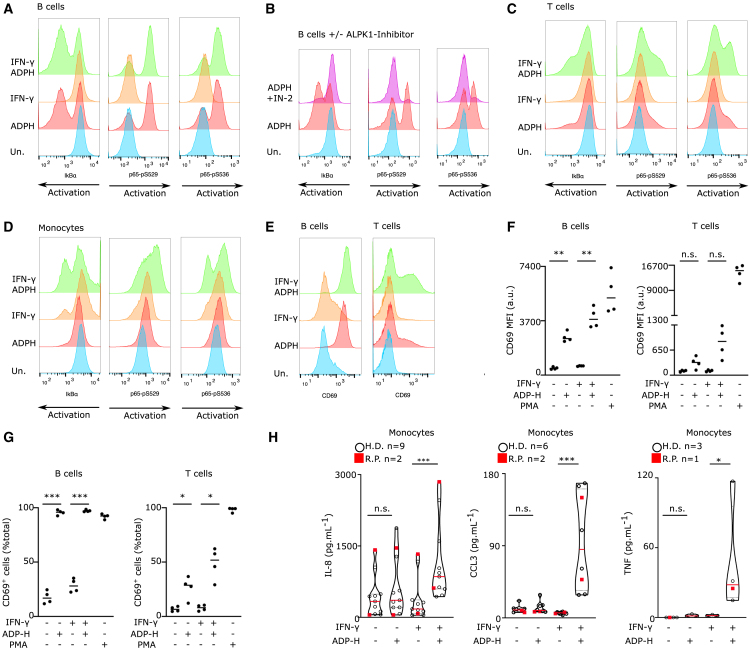
IFN-γ is required to license ADP-heptose responsiveness in monocytes while it is dispensable in B cells (A–D) PBMCs from 3 healthy donors (HD) were primed or not with IFN-γ for 16 h followed by 30 min stimulation with ADP-heptose (ADPH) at 10 μM. (B) When applicable, cells were pretreated with ALPK1 inhibitor (IN-2) at 10 μM, 30 min before addition of ADPH. IκBα (left panel), NF-κB p65 phosphoserine 529 (middle panel) and, NF-κB p65 phosphoserine 536 (right panel) were detected by flow cytometry following gating on (A-B) CD3ε^−^ HLA-DR^+^ CD20^+^ cells (B cells), (C) CD20^−^ CD3ε^+^ cells (T cells) and (D) CD20^−^ CD3ε^+^ HLA-DR^+^ cells (monocytes). (E–G) CD14^−^ cells from 4 healthy donors were primed or not with IFN-γ for 16 h followed by 6 h stimulation with ADP-heptose (ADPH) at 1 μM. CD69 was detected by flow cytometry following gating on CD3ε^−^ CD19^+^ cells (B cells, left panel) and, CD19^−^ CD3ε^+^ cells (T cells, right panel). (F) CD69 mean fluorescence intensity (MFI) is quantified (a.u., arbitrary units). (G) The percentage of CD69^+^ cells is shown. (H) Primary monocytes from HD (open circle, *n* = 9) or ROSAH syndrome patients (red square, n = 1–2) were primed or not with IFN-γ (1000 u/mL) for 16 h, and then treated by ADP-heptose (1 μM) for 6 h. IL-8, CCL3, and TNF were quantified in the supernatant by ELISA. (A–E) Concatenates from three healthy donors are shown. (F and G) One dot corresponds to the value from one healthy donor, the bar corresponds to the mean of 4 healthy donors. One Way ANOVA with Holm-Sidak correction was performed (F: ∗∗: *p* = 0.0032, F: ∗∗∗*p* < 0.001; ∗ (from left to right) *p* = 0.023, *p* = 0.017). (H) Friedman paired test with Dunn’s correction for multiple tests was performed (n.s.: *p* = 0.4954; ∗∗∗: *p* < 0.001). Each symbol represents the average value from a biological triplicate from one individual, the red line shows the median and the dotted lines the quartile.

ALPK1/TIFA pathway has primarily been investigated in epithelial cells,^1^^,^^6^^,^^7^^,^^15^^,^^17^^,^^18^^,^^19^^,^^20^^,^^21^ and knowledge regarding its regulation in human cells remains limited. Therefore, we evaluated NF-κB activation in PBMCs upon stimulation by ADP-heptose. Three parameters were examined using flow cytometry (Figure S1A): IκBα degradation and phosphorylation of NF-κB p65 at serine residues 529 and 536. Notably, B lymphocytes exhibited a clear response, with 60% displaying IκBα degradation and simultaneous p65 phosphorylation on the considered serine residues (Figure 1A). This activation was fully reversed upon pre-treatment with an ALPK1 inhibitor (Figure 1B) while PMA-mediated NF-κB activation was not statistically reduced (Figures S1B–S1E). Interestingly, this activation was not observed with LPS treatment (Figure S1I) suggesting that ADP-heptose is a specific activator of NF-κB in B cells. In contrast, only a very small proportion of T cells showed NF-κB activation upon ADP-heptose treatment (Figure 1C), while PMA treatment activated NF-κB in the entire population of both B and T cells (Figures S1F and S1G). LPS treatment did not promote NF-κB activation in T cells (Figure S1J). Monocytes exhibited minimal NF-κB activation upon ADP-heptose stimulation (Figure 1D) while LPS (but not PMA) induced a robust activation in these cells (Figures S1H and S1K). These results demonstrate that at steady state, primary human mononuclear cells exhibit different responsiveness to ADP-heptose.

IFN-γ is a potent priming agent in numerous innate immune signaling pathways,^22^^,^^23^^,^^24^ we thus investigated whether IFN-γ priming could enhance ADP-heptose responsiveness in monocytes and T lymphocytes. Indeed, upon IFN-γ priming, ADP-heptose treatment resulted in robust NF-κB activation in both monocytes (Figure 1D) and T lymphocytes (Figure 1C). However, IFN-γ priming did not substantially affect NF-κB activation in B lymphocytes (Figure 1A).

To confirm the differential responsiveness of PBMCs to ADP-heptose, we investigated the upregulation of the activation marker CD69 on the surface of lymphocytes 16 h post-ADP-heptose treatment, with or without IFN-γ priming (Figure S1L). This staining confirmed that B cells are constitutively highly responsive to ADP-heptose (Figures 1E–1G). IFN-γ priming substantially up-regulated CD69 in T lymphocytes in response to ADP-heptose, although the level of upregulation remained much lower than the one observed following PMA treatment (Figures 1E–1G and S1M). Interestingly, the increase in CD69 (both in terms of percentage of positive cells-Figure S1O and in terms of mean fluorescence intensity-Figure S1P) was higher in effector cells compared to the one observed in naive T cells. In addition, effector memory and to a lesser extent central memory T cells displayed a greater response to ADP-heptose in terms of CD69 upregulation than naive T cells did suggesting that antigen exposure sensibilizes T cell to ADP-heptose responsiveness (Figures S1O and S1P). IFN-γ priming itself induced CD69 upregulation on the monocyte surface (Figures S1N, S1Q, and S1R). Consequently, this marker could not be used in monocytes to study the impact of IFN-γ on ADP-heptose responsiveness.

The response of monocytes to ADP-heptose was thus monitored by quantifying IL-8, CCL3 and TNF secretion in the presence or absence of IFN-γ priming (Figure 1H). In the absence of IFN-γ priming, ADP-heptose did not elicit a cytokine/chemokine response. Conversely, upon IFN-γ priming followed by ADP-heptose treatment, monocytes significantly released IL-8, CCL3 and TNF. Of note, B cells despite their responsiveness to ADP-heptose did not produce substantial amount of IL-8 (Figure S1S), suggesting that, at least for a subset of cytokines, the systemic response to ADP-heptose may be mostly driven by monocytes/macrophages.

Altogether, these results demonstrate that, at steady state, different human PBMCs subtypes exhibit different responsiveness to ADP-heptose. Particularly, IFN-γ priming is required to license the ALPK1 pathway in primary human monocytes but not in B cells, that are, at steady state, already highly responsive to ADP-heptose.

### IFN-γ induces ALPK1 and TIFA in monocytes

**Figure 2:**
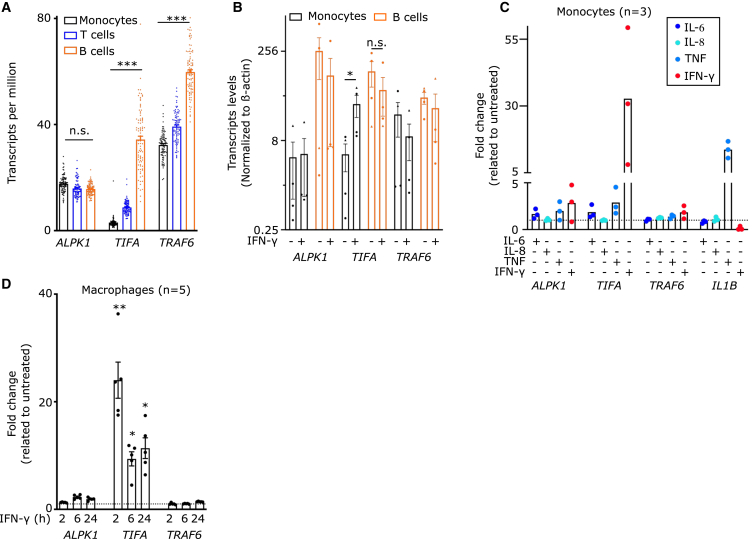
*TIFA* is differentially expressed at steady state in B cells and monocytes and strongly induced in the latter cells by IFN-γ (A) *ALPK1*, *TIFA* and *TRAF6* transcript levels in monocytes (black), T cells (blue) or B cells (orange) were obtained from the public RNA-seq dataset DICE. (B) Transcript level of the indicated gene was determined by qRT-PCR in primary human monocytes (black) or B cells (orange) treated or not for 16 h with IFN-γ and normalized to β-actin levels. (D) Primary monocytes-derived macrophages from 5 healthy donors were treated with IFN-γ for the indicated time. *ALPK1*, *TIFA* and *TRAF6* transcript levels were analyzed by RNA-seq and normalized to the untreated sample. (C) Transcript level of the indicated gene was determined by qRT-PCR in primary human monocytes treated for 16 h with IL-6 (100 ng/mL), IL-8 (100 ng/mL), TNF (100 ng/mL) or IFN-γ (1000 u/mL), normalized to β-actin levels and to the untreated sample. (A) Kruskal-Wallis unpaired test with Dunn’s correction for multiple tests was performed (n.s.: *p* = 0.22; ∗∗∗: *p* < 0.001). Each dot represents the value from one healthy donor, the bar represents the mean ± SEM. (B) Each dot and triangle represent the average value from a technical duplicate from one healthy donor (*n* = 3) or one ROSAH syndrome patient (*n* = 1), respectively. The bar represents the mean ± SEM. Friedman paired test with Dunn’s correction for multiple tests was performed (∗: *p* = 0.037, n.s.: *p* > 0.99). (C) Each dot represents the average value from a technical triplicate from one healthy donor (*n* = 3). The bar represents the mean. (C and D) The dotted horizontal line (y = 1) indicates an absence of change in transcript levels compared to untreated. (D) Each dot represents the value from one individual, the bar represents the mean ± SEM. Friedman paired test with Dunn’s correction for multiple tests was performed (∗∗: *p* = 0.006, ∗: *p* = 0.04, *p* = 0.03 from left to right, respectively).

To explore the basis of the differential regulation of the ALPK1 pathway in human PBMCs, we analyzed an RNA-seq dataset of PBMCs obtained from healthy donors.^25^ At steady state, *TIFA* expression in monocytes was minimal, while its expression level was more than 10-fold higher in B cells (Figure 2A).

An intermediate *TIFA* level was observed in T cells (Figure 2A) with higher level in memory cells compared to naive T cells (Figure S2A). *TRAF6* was also expressed at significantly higher levels in B cells than in monocytes, but the steady state quantities of *TRAF6* transcripts in the latter cells were much higher than those of *TIFA*. In contrast, *ALPK1* was expressed at similar levels in monocytes, T cells, and B cells (Figure 2A). On the other hand, *TLR4*, *MYD88*, *NOD2*, and *RIPK2* were significantly more expressed in monocytes than in naive B cells (Figure S2B). The expression pattern of *TIFA* (higher in B cells than in monocytes) thus appears largely specific among the innate immune genes leading to NF-κB activation (apart from *NOD1,* which is expressed at a 2-fold higher level in naive B cells than in monocytes).

The higher expression level of *TIFA* in B cells compared to monocytes was confirmed by qRT-PCR (Figure 2B). Interestingly, upon IFN-γ treatment, *TIFA* transcript levels significantly increased in monocytes, reaching levels similar to the ones observed in B cells. In contrast, neither *ALPK1* nor *TRAF6* transcript levels were affected by IFN-γ treatment in monocytes. IFN-γ treatment did not significantly modulate *TIFA* transcript levels in B cells. IL-6 and TNF (but not IL-8 which receptor is not expressed in monocytes) induced a moderate upregulation of *TIFA* transcript level in monocytes although one order of magnitude lower than the induction observed after IFN-γ treatment (Figure 2C).

IFN-γ-mediated regulation of *TIFA* transcript level was also observed in primary human macrophages. Indeed, *TIFA* transcript levels increased strongly and rapidly following IFN-γ treatment (Figure 2D). A small increase (2.3-fold) in *ALPK1* levels was observed at 6 h post IFN-γ treatment while *TRAF6* levels were not modified. Engagement of TLR1/2 by a bacterial lipopeptide also induced *TIFA* expression in these cells although more transiently and to a lower extent than IFN-γ (Figure S2C).

These results indicate that *TIFA* transcript levels correlate with the responsiveness of the various human PBMCs to ADP-heptose at steady state. Furthermore, these findings suggest that *TIFA* transcript level may represent the limiting factor regulated by IFN-γ (and to a lesser extent by NF-κB-activating stimuli) in monocytes (and possibly macrophages) to license ADP-heptose-responsiveness.

### TIFA induction is sufficient to explain the IFN-γ -mediated licensing effect

**Figure 3:**
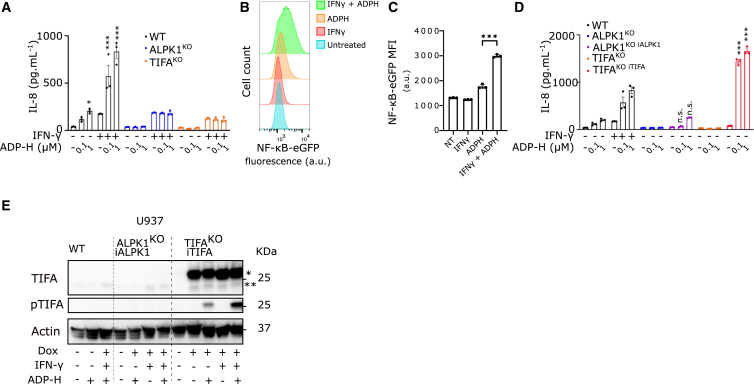
IFN-γ induces *TIFA* expression to license ADP-heptose responsiveness in monocytes (A) U937 WT (black), *ALPK1*^KO^ (blue) or *TIFA*^KO^ (orange) were primed with IFN-γ and stimulated for 6 h with ADP-heptose (ADP-H) at the indicated concentration. IL-8 in the supernatant was quantified at 6 h post-treatment. (B and C) U937 cells expressing eGFP under the control of an NF-κB responsive promoter were primed or not with IFN-γ and treated with ADP-heptose (100 nM) for 6 h. GFP intensity was measured by flow cytometry. Histograms are shown in (B) and mean fluorescence intensity (MFI) is shown in (C). (D) U937 WT (black), ALPK1^KO^ (blue), ALPK1 ^KO^ complemented with ALPK1 under the control of a doxycycline-inducible promoter (ALPK1^KO iALPK1^ (purple)), TIFA^KO^ (orange) or TIFA^KO^ complemented with TIFA under the control of a doxycycline inducible promoter (TIFA^KO iTIFA^ (red)) were treated with doxycycline, primed or not with IFN-γ and stimulated for 6 h with ADP-heptose (ADP-H) at the indicated concentration. IL-8 in the supernatant was quantified at 6 h post-treatment. (E) U937 WT, ALPK1 ^KO^ complemented with ALPK1 under the control of a doxycycline-inducible promoter (ALPK1^KO iALPK1^), TIFA^KO^ complemented with TIFA under the control of a doxycycline inducible promoter (TIFA^KO iTIFA^) were treated as indicated with doxycycline, primed or not with IFN-γ and stimulated for 30 min with ADP-heptose (ADP-H) at 1 μM and analyzed by western blot. ∗ indicates 3X-Flag TIFA while ∗∗indicates endogenous TIFA protein. (A) Each dot represents one biological replicate; the bar represents the mean ± SEM from three replicates. One experiment representative of three independent experiments is shown. One-way Anova with Sidak’s correction for multiple tests was performed ∗: *p* = 0.012, ∗∗∗: *p* < 0.001. (C and D) a.u. arbitrary units. One-way ANOVA with Sidak’s correction for multiple tests was performed. ∗∗∗: *p* < 0.001. Each dot represents one biological replicate, the bar represents the mean ± SEM from three replicates. One experiment representative of three independent experiments is shown. (D) One-way ANOVA with Sidak’s correction for multiple tests was performed to compare to IL-8 values obtained in WT U937 treated with ADP-heptose in the absence of IFN-γ. n.s.: 1.00, ∗∗∗: *p* < 0.001.

To explore the molecular bases of this regulation, we employed U937 cells, a genetically-modifiable human monocytic cell line. This cell line presents *ALPK1* and *TIFA* transcript levels similar to the ones present in primary human monocytes (Figures S3A and S3B). IFN-γ induces both *ALPK1* and *TIFA* transcript upregulation in U937 cells (Figures S3A and S3B). Consistent with observations in primary human monocytes, IFN-γ augmented ADP-heptose-mediated IL-8 secretion in WT U937 cells (Figure 3A). This response was absent in *ALPK1*^KO^ and *TIFA*^KO^ U937 cells, demonstrating the specificity of the studied mechanism (Figures 3A, S3C, and S3D).

Although less potent than IFN-γ, both IFN-α2 and IFN-β significantly increased IL-8 secretion in response to ADP-heptose (Figure S3E). The licensing effect of IFN-γ was also confirmed in U937 cells expressing eGFP under the control of an NF-κB-responsive promoter (Figures 3B and 3C). Having validated this experimental system, we next assessed whether upregulation of ALPK1 or TIFA could explain the IFN-γ-mediated regulation of ADP-heptose responsiveness observed in monocytes. To control ALPK1 and TIFA expression independently of IFN-γ, we cloned *ALPK1* and *TIFA* cDNA under a doxycycline-inducible promoter. qRT-PCR analyses confirmed that in the *ALPK1*^KO^ cell lines transduced with the doxycycline-inducible *ALPK1* plasmid (iALPK1) and in the *TIFA*^KO^ cell line transduced with the doxycycline-inducible *TIFA* plasmid (iTIFA), the expression level of *ALPK1* and *TIFA* transgene was independent of IFN-γ (Figure S3F). Furthermore, Western blot analyses showed that endogenous TIFA protein levels were inducible by IFN-γ, and that doxycycline treatment allowed TIFA expression independently of IFN-γ (Figure 3E).

Importantly, doxycycline-mediated expression of *TIFA* (in the TIFA^KO^ iTIFA cell line) was sufficient to promote a robust IL-8 secretion in response to ADP-heptose, in the absence of IFN-γ (Figure 3D). In contrast, ectopic expression of *ALPK1* did not significantly enhance ADP-heptose responsiveness, demonstrating that IFN-γ-mediated regulation of the pathway lies mostly in the transcriptional regulation of *TIFA* levels.

Ectopic expression of *TIFA* was sufficient to promote TIFA phosphorylation in response to ADP-heptose (Figure 3E). Interestingly, in the presence of IFN-γ, we observed an increase in ADP-heptose-induced phosphorylation of TIFA in the TIFA^KO^ iTIFA cell line. This result indicates that while *TIFA* induction by IFN-γ is sufficient to license ADP-heptose responsiveness in monocytes, other IFN-inducible factors may contribute to further potentiate the ADP-heptose response.

### IFN-γ promotes phenotypic expression of ALPK1 pathogenic variants in monocytes

Several ALPK1 variants have been associated with ROSAH syndrome,^8^^,^^12^ spiradenoma, and spiradenocarcinoma,^14^ as well as PFAPA syndrome.^16^

**Figure 4:**
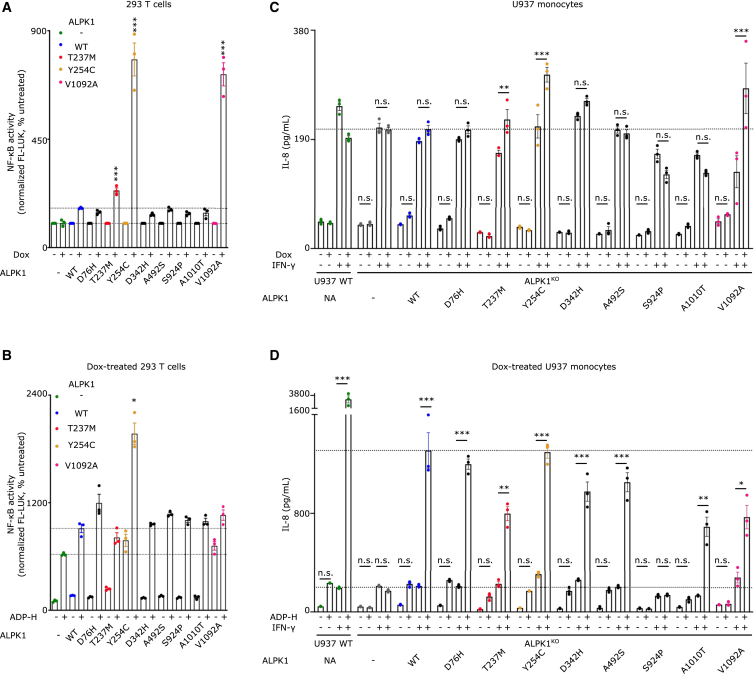
IFN-γ triggers ALPK1 pathogenic variants phenotype expression in monocytes (A and B) 293T were transfected with the plasmid encoding the indicated doxycycline-inducible ALPK1 variants or PSTPIP1 as a control (−) together with a plasmid encoding the firefly-luciferase under the control of an NF-κB-responsive promoter and a plasmid encoding the renilla-luciferase under a constitutive promoter. Cells were exposed or not to doxycycline for 16 h before measurement of luciferase. Firefly luciferase signal was normalized to renilla luciferase signal to obtain NF-κB activity. NF-κB activity in the absence of doxycycline was normalized to 100% for each cell line. (B) Doxycycline-induced cells were treated or not with ADP-heptose (1 μM) for 3 h before measurement of luciferase signals. (C and D) U937 monocytes WT, ALPK1^KO^ or ALPK1^KO^ complemented with the indicated doxycycline-inducible ALPK1 variants were treated with doxycycline, IFN-γ for 16 h and (D) ADP-heptose at 1 μM for 6 h. IL-8 release in the supernatant was quantified by ELISA. (A-D) Each dot represents one biological replicate, the bar represents the mean ± SEM from three replicates. One experiment representative of three independent experiments is shown. (A) One-way ANOVA with Sidak’s correction for multiple tests was performed to compare the normalized NF-κB activity obtained with the indicated variant to the one obtained in cells transfected with WT ALPK1. ∗∗∗: *p* < 0.001. Dotted lines indicate the 100% level and the normalized NF-κB activity obtained upon WT ALPK1 expression. (B) Kruskal-Wallis test with Dunn’s correction for multiple tests was performed to compare the normalized NF-κB activity obtained with the indicated ALPK1 variant upon ADP-heptose treatment to the one obtained in cells transfected with WT ALPK1 and treated with ADP-heptose. ∗: *p* = 0.027. Dotted lines indicate the normalized NF-κB activity obtained upon ADP-heptose in the absence (endogenous ALPK1 expression) and presence of WT ALPK1 transfection. (C and D) One-way ANOVA with Sidak’s correction for multiple tests was performed. (C) ∗∗: *p* = 0.0013, ∗∗∗: *p* < 0.001. (D) ∗∗∗: *p* < 0.001; ∗∗: *p* = 0.0015; *p* = 0.0022 from left to right, respectively; ∗: *p* = 0.012.

We first assessed whether constitutive activation could be detected upon transient transfection of doxycycline-inducible ALPK1 variants in 293T cells expressing a luciferase-based NF-κB reporter (Figure S4A). As previously demonstrated by others, the two ROSAH syndrome variants and the recurrent spiradenoma variants led to a strong activation of NF-κB. No constitutive activation of NF-κB was observed upon the expression of PFAPA variants (p.D342H and p.S924P) or sporadic spiradenocarcinoma variants (p.D76H, p.A492S, p.A1010T) (Figure 4A). Notably, the mutations observed in the two PFAPA variants may destabilize the corresponding proteins, as they were not detected by western blot analysis upon expression in 293T cells (Figure S4A). At the transcript level, all constructs were inducible by doxycycline (Figure S4B). All variants responded to ADP-heptose, although to slightly different extent (Figure 4B).

We then complemented the *ALPK1*^KO^ U937 cell line with the different variants using lentivirus-mediated transduction and assessed IL-8 production upon variant induction (Figure 4C). In this experimental setting, which leads to lower levels of transgene expression than in the above 293T cells, no constitutive activation was detected upon doxycycline-mediated induction of ALPK1 variants (Figure 4C). We tested whether IFN-γ-mediated regulation of the pathway could reveal pathogenic variants. IFN-γ, by itself, induced a low level of IL-8. Interestingly, three cell lines expressing the pathogenic ROSAH and spiradenocarcinoma variants demonstrated a significant increase in IL-8 production upon doxycycline-mediated induction of the variants, but only in the presence of IFN-γ (Figure 4C). This result suggests that IFN-γ-mediating licensing of the ALPK1/TIFA pathway might contribute to autoinflammation in the absence of an exogenous ligand.

Importantly, even in cell lines expressing pathogenic variants, IFN-γ was required to promote IL-8 release in response to ADP-heptose (Figure 4D). The cell line expressing the p.S924P variant did not respond to ADP-heptose, likely due to the protein instability observed by Western blot (Figure S4A). The licensing effect of IFN-γ was also confirmed in primary human monocytes from ROSAH syndrome patients displaying the *ALPK1* p.T237M variant. ADP-heptose-dependent IL-8, CCL3 and TNF secretions were increased in the supernatant of monocytes from ROSAH syndrome patients in the presence of IFN-γ (Figure 1H). Of note, no secretion of CCL3 or TNF was observed in cells from these patients in the absence of both IFN-γ and ADP-heptose. Similarly, the constitutive secretion of IL-8 observed at steady state was within the range of that observed in healthy individuals (Figure 1H).

### JAK inhibitors block the licensing effect of IFN-γ on ADP-heptose responsiveness

**Figure 5:**
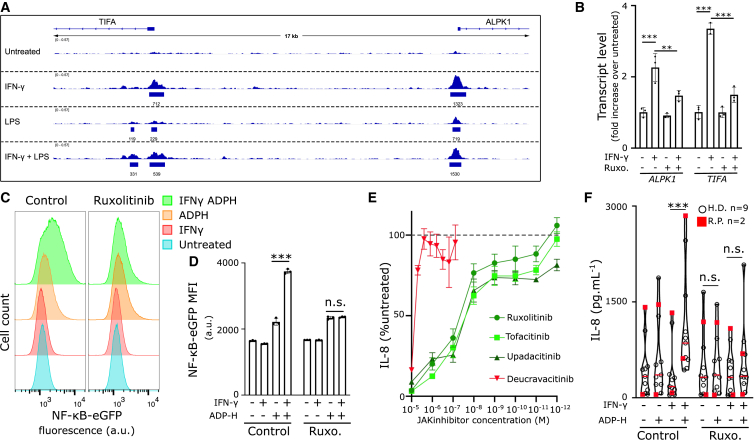
JAK inhibitors block IFN-γ-mediated ALPK1 responses (A) STAT1 Chip-seq data (GSE 43036) from primary human monocytes from one healthy donor treated or not with IFN- γ (100 u/mL) for 24 h and/or when indicated LPS (500 ng/mL) for 3 h were analyzed on the *TIFA*/*ALPK1* genomic region using ChIP-Atlas and integrative Genomics Viewer. The raw CHIP-seq and the peak-call data (q < 1E-05) are shown for each sample. MACS2 score is indicated under each detected peak. (B) *ALPK1*, and *TIFA* transcript levels in U937 cells treated as indicated with IFN- γ (100 u/mL) for 16 h in the presence or not of Ruxolitinib (Ruxo.) at 1 μM. (C and D) U937 expressing eGFP under the control of an NF-κB-responsive promoter were treated with IFN- γ (1,000 u/mL) for 16 h in the presence or not of Ruxolitinib (Ruxo.) at 1 μM followed by ADP-heptose (100 nM) treatment for 6 h. (C) flow cytometry histograms corresponding to a concatenate of three independent samples are shown. (D) Quantification of the mean fluorescence intensity (MFI) is shown. (E) U937 cells were treated with IFN- γ (1,000 u/mL) for 16 h in the presence of the indicated JAK inhibitor at the indicated concentrations. Cells were treated with ADP-heptose (1 μM) for 3 h and IL-8 concentration in the supernatant was quantified by ELISA. Results were normalized to IL-8 concentrations obtained in U937 treated with IFN- γ and ADP-heptose in the absence of JAK inhibitors. (F) Primary monocytes from HD (open circle, *n* = 9) or ROSAH syndrome patients (red square, *n* = 2) were primed or not with IFN-γ (1000 u/mL) for 16 h in the presence of Ruxolitinib (1 μM) as indicated, and then treated by ADP-heptose (1 μM) for 6 h. IL-8 was quantified in the supernatant by ELISA. (B, D, and E) Each dot represents the value from one sample, the bar represents the mean ± SEM. (B, D) One-way ANOVA with Sidak’s correction for multiple tests was performed. ∗∗∗: *p* < 0.001; ∗∗: *p* = 0.0047; n.s.: not statistically significant. (B–E) One experiment representative of 2 independent experiments is shown. (F) Friedman paired test with Dunn’s correction for multiple tests was performed (∗∗∗: *p* < 0.001, n.s.:>0.99; n.s. = 0.056, from left to right). Each symbol represents the average value from a biological triplicate from one individual, the red line shows the median and the dotted lines the quartile.

The impact of IFN-γ on the ALPK1/TIFA pathway implies a role for the JAK/Stat pathway downstream of the IFN-γ receptor. Accordingly, ChIP-seq analyses revealed specific binding of STAT1 to both the *TIFA* and *ALPK1* promoters in primary human monocytes in the presence of IFN-γ (Figure 5A). LPS treatment also resulted in increased STAT1 binding, possibly due to the ability of LPS to induce type I IFNs and the presence of STAT1 downstream of the type I IFN receptor.

We thus decided to assess whether Ruxolitinib, a JAK1/2 inhibitor, could block the licensing effect of IFN-γ on ADP-heptose responsiveness in monocytes. We first observed that Ruxolitinib significantly inhibited IFN-γ-mediated upregulation of *ALPK1* and *TIFA* transcripts in U937 cells (Figure 5B). Subsequently, using the above described U937 cell line expressing the fluorescent NF-κB reporter, we demonstrated that Ruxolitinib abolished IFN-γ/ADP-heptose-mediated NF-κB activation (Figures 5C and 5D).

Finally, we monitored IL-8 secretion in IFN-γ-primed U937 cells treated with ADP-heptose in the presence of various JAK inhibitors to test their potential to inhibit IFN-γ/ADP-heptose-mediated IL-8 release (Figure 5E). Despite different spectra of suggested selectivity against JAK family members,^26^ Ruxolitinib, Tofacitinib, and Upadacitinib all inhibited IL-8 response with similar efficiency. In contrast, Deucravacitinib, with suggested selectivity for TYK2 (a JAK downstream of type I IFN receptor among others), demonstrated a lower ability to inhibit IL-8 release.

Finally, the inhibition potential of Ruxolitinib was tested on primary human monocytes from healthy donors and two ROSAH syndrome patients. In either case, Ruxolitinib blocked IFN-γ/ADP-heptose-mediated IL-8 release (Figure 5F). A similar efficiency of Ruxolitinib was observed when monitoring CCL3 (Figure S5A) or TNF (Figure S5B) release.

Altogether, these results indicate that several JAK inhibitors could be used to block the licensing effect of IFN-γ on NF-κB activation and its downstream consequences.

## Discussion

The ALPK1/TIFA pathway is the latest human innate immune pathway discovered, and its regulation is still incompletely understood. Here, we uncovered that this pathway is differentially regulated in various human blood mononuclear cells, with a constitutive ability to sense ADP-heptose in B cells, while monocytes require IFN-γ priming to license this pathway. The reason why the innate immune system has evolved such differential regulation remains an open question, but one can speculate that additional control mechanisms are necessary in monocytes before triggering NF-κB activation and the release of numerous cytokines and chemokines that orchestrate downstream systemic responses.

The consequences of physiological and pathogenic ALPK1 signaling in B cells remain unclear. Thirty-eight percent of the studied ROSAH patients (5/13) had low IgM concentrations.^12^ Lymphopenia was reported in 41% of patients but did not appear to be specific of B cells.^12^ Hecker and colleagues reported an almost complete absence of B cells in a ROSAH patient, although a treatment with Rituximab (anti-CD20 targeting B cells) administered 2 years before the analysis might have contributed to this depletion.^10^ The impact of TLRs signaling in B cell tolerance and in the removal of autoreactive B cells is well known.^27^^,^^28^ Since TLRs and ALPK1 share downstream effectors, it is tempting to speculate that the ALPK1/TIFA pathway may impact B cell functions. Further studies are needed to study the impact of ALPK1 and of its gain and loss of function variants in B cells.

Our study extends the number of innate immune signaling pathways under the direct regulation of IFN-γ. Indeed, nucleotide-binding oligomerization domain containing 2 (NOD2) is strongly inducible by IFN-γ in macrophages, and IFN-γ triggers NF-κB activation in macrophages from Blau syndrome patients.^29^ Similarly, IFN-γ increases the expression of the inflammasome sensor pyrin and licenses the activation of the pyrin inflammasome in human monocyte-derived macrophages.^30^ Importantly, IFN-γ priming of monocytes from PAPA (pyogenic arthritis, pyoderma gangrenosum and acne) syndrome patients induces a pyrin-dependent IL-18 release and JAK inhibitors have a beneficial effects on the skin lesions of these patients.^31^ The inclusion of the ALPK1/TIFA pathway in the large repertoire of IFN-γ-regulated immune responses is highly consistent with the strong anti-bacterial role of IFN-γ and the ability of ALPK1 to sense bacterial nucleotide sugars. Interestingly, IFN-γ is a well-known protective cytokine in numerous infections including during infections with bacteria (e.g., *Neisseria gonorrhoeae*^32^ or *Burkholderia cenocepacia*^33^) well characterized for their ability to activate the ALPK1/TIFA pathway.^1^^,^^21^ Alternatively, IFN-γ may also contribute to the deleterious inflammation observed during various Gram-negative infections^34^ possibly in part by promoting pro-inflammatory responses to ADP-heptose-releasing bacteria (e.g., *Campylobacter jejuni* during colitis^35^^,^^36^ or *Neisseria meningitidis* during meningitis^19^^,^^37^).

IFN-γ induces expression of numerous genes. TIFA appears to be the key limiting protein in the signaling pathway in monocytes. Following phosphorylation, TIFA oligomerizes to promote TRAF6 oligomerization; the number of TIFA molecules within a cell thus likely controls the ability to form large oligomers, known as TIFAsomes, which can act as supramolecular organizing centers^38^ to activate downstream signaling events. In contrast, modulation of ALPK1 levels did not significantly affect NF- kB activation in our experimental system, possibly because ALPK1 is an enzyme, and minute amounts of the protein are sufficient to promote downstream events. Interestingly, TIFA levels appear to result from the integration of several transcriptional and post-transcriptional regulations. In addition to the potent IFN-γ-mediated regulation identified in this study, *TIFA* transcription is upregulated following NF-κB activation.^20^^,^^39^ Conversely, following activation, TIFA is ubiquitinated and degraded in a proteasome and lysosome-dependent manner.^39^

Most of the IFN-γ-mediated regulation of the ALPK1-TIFA pathway appears to lie in the regulation of *TIFA* transcript levels (Figure 3D). Yet, we observed an increase in TIFA phosphorylation in *TIFA*^KO^iTIFA U937 cells ectopically expressing TIFA under the control of a doxycycline-inducible promoter (Figure 3E). This observation suggests that an upstream step of the pathway is also positively controlled by IFN-γ. Induction of *ALPK1* mRNA by IFN-γ may explain this result (Figures 2B–2D and 5B), although we cannot exclude other potential mechanisms, including the regulation of ADP-heptose transport into the host cell by IFN-γ.

ROSAH syndrome has been recently recognized as an autoinflammatory syndrome.^9^^,^^10^^,^^12^ Here, we demonstrate that IFN-γ licensing is required in monocytes from both healthy individuals and ROSAH disease patients. The transcriptional regulation of *TIFA* thus appears normal in patients with ROSAH syndrome. Using U937 cell lines to model monocytes expressing various ALPK1 variants, we observed that IFN-γ is promoting IL-8 release specifically in cells expressing either one of the two ROSAH variants (p.T237M or p.Y254C) or in cells expressing the recurrent spiradenoma variant (p.V1092A), suggesting that IFN-γ can functionally promote expression of the pathogenic trait. In line with this hypothesis, increased expression of interferon-regulated genes and phosphorylation of STAT1 have been described in patients with ROSAH syndrome.^12^ Deregulation of NF-κB in cell expressing pathogenic ALPK1 variants is thought to be due to the detection of self-nucleotide sugars. It remains to be demonstrated whether this response to self-nucleotide sugars in monocytic cell lines requires IFN-γ and whether differences in self-nucleotide sugars concentrations in different cell types may promote cell-specific responses in the context of *ALPK1* mutation.

The role of IFN-γ and of the JAK/STAT pathway demonstrated here *in vitro* hints for a therapeutic potential of JAK inhibitors to treat ROSAH patients, and potentially the deleterious oncogenic ALPK1/TIFA-dependent inflammation.^6^^,^^14^ Similarly, Tofacitinib has been proposed as a potential therapeutic agent for Blau syndrome.^40^ Interestingly, anti-IL-6 (Tocilizumab) improved intraocular inflammation in two ROSAH patients.^12^ Since IL-6 signals through JAK1/2, JAK inhibitors might be beneficial through a dual action on both IFN-γ and IL-6 signaling. The efficiency of JAK inhibitors on the different symptoms remains to be tested, but JAK inhibitors may provide a safe alternative to Tocilizumab in waiting for a specific drug targeting ALPK1.

### Limitations of the study

We were not able to observe a significant difference in cytokine secretion between IFN-γ-treated monocytes from healthy donors and ROSAH disease patients (Figure 1H), possibly due to the large interindividual variability in cytokine secretion, to the low number of ROSAH patients included in this study and to the fact that some of these patients were under treatment. In addition, one limitation of our study is that it remains unknown whether the systemic inflammation observed in ROSAH patients is due to a direct action of ALPK1 in myeloid cells or in other cell types (e.g., in the eye or in the sweat glands), in which the IFN-γ-mediated regulation of TIFA may be less important.

## Resource availability

### Lead contact

Requests for further information and resources should be directed to and will be fulfilled by the lead contact, Thomas Henry Thomas.henry@inserm.fr.

### Material availability

Plasmids generated in this study are being deposited to Addgene, please check https://www.addgene.org/Thomas_Henry/.

All the resources described in this paper are available upon reasonable request to the lead contact.

### Data and code availability

#### Data

This paper analyzes existing, publicly available data, accessible at https://dice-database.org, https://www.proteinatlas.org. Public ChipSeq data was from GSE 43036.

Full Western blot images are available at Mendeley https://doi.org/10.17632/hb5t3r68yh.1.

#### Code

This paper does not report original code.

#### Additional information

Any additional information required to reanalyze the data reported in this paper is available from the lead contact upon reasonable request.

## Acknowledgments

We would like to thank Peter Steinberger, Kosuke Yusa, Didier Trono, Stephen J. Elledge, Pierre-Olivier Vidalain for providing plasmids. The authors thank all the patients who took part in this study and Christine Berthet for her help in coordinating the clinical study at APHP. We acknowledge the contribution of the Etablissement Français du Sang Auvergne - Rhône-Alpes, and the contribution of SFR Biosciences (UMS3444/CNRS, US8/Inserm, ENS de Lyon, UCBL) Anira cytometry and protein Science facilities. We thank Flora Magnotti and I2BA team for fruitful discussions along the project. This work was funded by 10.13039/501100001677Inserm, 10.13039/501100001665Agence Nationale de la Recherche (ANR-21-CE15-0040). E.G. was funded by Année Recherche (ARS Auvergne-Rhône-Alpes).

## Author contributions

A.M., S.C., E.G., and P.B. performed the experiments; Y.J., N.M., G.M., and D.S. provided substantial clinical inputs on the work; Y.J. and N.M. obtained the ethical approval. Y.J. and T.H. designed and interpreted the work. T.H. wrote the manuscript; all authors reviewed and approved the manuscript.

## Declaration of interests

T.H. team obtained a research contract with financial support from Drug Farm, a company that develops agonists and inhibitors of ALPK1. Y.J. received consultant fees from Drug Farm. Other authors did not report any conflict of interest related to the present work.

## Declaration of generative AI and AI-assisted technologies in the writing process

During the preparation of this work the authors used ChatGPT to proofread the manuscript. After using this tool/service, the authors reviewed and edited the content as needed and take full responsibility for the content of the publication.

## STAR★Methods

### Key resources table

**Table:** 

REAGENT or RESOURCE	SOURCE	IDENTIFIER
**Antibodies**		

IkB-alpha-PE	BD Biosciences	Cat#560818; RRID: AB_2033975
P65-pS529-AF647	BD Biosciences	Cat#558422; RRID: AB_647136
P65-pS536-AF488	Cell Signaling Technology	Cat#4886; RRID: AB_390789
CD69-AF488	BD Pharmingen	Cat#567399; RRID:
CD20-PE-Cy7	BD Biosciences	Cat#561175; RRID: AB_10562032
CD3e-V450	BD Biosciences	Cat#560365; RRID: AB_1645570
HLA-DR-BUV395	BD Biosciences	Cat#564040; RRID: AB_2738558
CD19-PE	Biolegend	Cat#363004; RRID: AB_2564126
CD14-APC	Biolegend	Cat#325608; RRID: AB_830681
IL-8-PE	BD Biosciences	Cat#554720; RRID: AB_395529
ALPK1	Abcam	Cat#Ab236626
TIFA	Abcam	Cat#Ab239352
b-actin	Sigma-Aldrich	Cat#MAB1501; RRID: AB_2223041
PhosphoT9-TIFA	Abcam	Cat#Ab214815
CD127-BV785	BioLegend	Cat#351329; RRID: AB_11219610
CD8-BV650	BioLegend	Cat#344729; RRID: AB_2564509
CD45RA- PE-Cy7	BioLegend	Cat#304125; RRID: AB_10709440
CD4-APC	BioLegend	Cat#317415; RRID: AB_571944
CD197-APC-Cy7	BioLegend	Cat#353211; RRID: AB_10915272
CD25-Spark PLUS UV395	BioLegend	Cat#385609; RRID: AB_3106236

**Bacterial and virus strains**		

*Escherichia coli* NEB 5-alpha	NEB	Cat#C2987H
*Escherichia coli* DB3.1	Thermofisher scientific	Cat#11782-018

**Biological samples**		

Healthy adult blood tubes	Etablissement Français du Sang (EFS)	Cat#T3001
Healthy adult blood pack	Etablissement Français du Sang (EFS)	Cat#A3101

**Chemicals, peptides, and recombinant proteins**		

Lymphocyte Separation Medium	Eurobio	Cat#CMSMSL01-01
CD14 MicroBeads, human	Miltenyi Biotec	Cat#130-050-201
CD19 MicroBeads, human	Miltenyi Biotec	Cat#130-050-301
Propidium iodide	Sigma-Aldrich	81845; CAS: 25535-16-4
Recombinant Human Interferon Gamma (rh IFN-gamma)	Immunotools	Cat#11343537
ADP-Heptose	Invivogen	Cat#tlrl-adph-l
Phorbol myristate acetate - (PMA)	Invivogen	tlrl-pma; CAS: 16561-29-8
LPS-EB Ultrapure	Invivogen	Cat#tlrl-3pelps
Perm Buffer II	BD-Bioscience	558052; CAS: 67-56-1
Perm/Wash Buffer	BD Biosciences	Cat#554723
BD GolgiStop™ Protein Transport Inhibitor	BD Biosciences	Cat#554724
BD Cytofix/Cytoperm™ solution	BD Biosciences	Cat#554722; RRID: AB_2869010
BD Perm/Wash™ Perm/Wash Buffer	BD Biosciences	Cat#554723
IFN alpha 2b, human recombinant	Biovision	Cat#4595-100
Recombinant Human Interferon-beta 1 alpha (rh IFN-beta 1a)	Immunotools	Cat#11343520
Recombinant Human Interleukin 6 (rh IL-6)	Immunotools	Cat#11340064
Recombinant Human Interleukin-8/1-77a.a. (rh IL-8/1-77a.a.)	Immunotools	Cat#11349084
Recombinant Human Tumor Necrosis Factor-alpha (rh TNF-alpha)	Immunotools	Cat#11343015
ALPK1-IN-2	MedChemExpress	HY-147562; CAS: 2765633-68-7
Ruxolitinib	Selleckchem	SE-S1378; CAS: 941678-49-5
Tofacitinib	MedChemExpress	HY-40354; CAS: 477600-75-2
Deucravacitinib	MedChemExpress	HY-117287; CAS: 1609392-27-9
Upadacitinib	MedChemExpress	HY-19569; CAS: 1310726-60-3
Puromycin (solution)	Invivogen	ant-pr-1; CAS: 58-58-2
G418 disulfate salt (geneticin)	Sigma-Aldrich	A1720; CAS: 108321-42-2
Doxycycline hyclate	Sigma-Aldrich	D9891; CAS: 24390-14-5
cOmplete, EDTA-free Protease Inhibitor Cocktail Tablets	Roche	Cat#05056489001
Sodium fluoride	Sigma-Aldrich	S1504; CAS: 7681-49-4
4-15% Mini-PROTEAN TGX Precast Protein Gels, 15-well, 15 μL	Bio-Rad	Cat#4561086
Trans-Blot Turbo RTA Transfer Kit, Midi, PVDF, for 40 Blots	Bio-Rad	Cat#3727660
Separation Module, 8 × 25 capillary cartridges 12–230 kDa	Bio-techne	Cat#SM-W004
Random primers	Promega	Cat#C1181
OligodT primers	Promega	Cat#C1101
FuGENE® HD Transfection Reagent	Promega	E2311; CAS: 64-17-5
TRI Reagent	Sigma-Aldrich	Cat#T9424
7-AAD Viability Staining Solution	BioLegend	Cat#420403

**Critical commercial assays**		

Gateway LR Clonase Enzyme mix	ThermoFisher Scientific	Cat#11791019
QuikChange II XL site-directed	Agilent	Cat#200522
Nucleospin RNA kit	Macherey Nagel	Cat#740955
ImProm-II™ Reverse Transcription System	Promega	Cat#A3802
FastStart Universal SYBR Green Master Mix	Sigma-Aldrich	Cat#4913914001
BD Cytofix/Cytoperm™ Fixation/Permeabilization Kit	BD Biosciences	Cat#554714
Dual-Glo® Luciferase Assay System	Promega	Cat#E2940
Human IL-8/CXCL8 DuoSet ELISA	Bio-techne	Cat#DY208
Human CCL3/MIP-1 alpha DuoSet ELISA	Bio-techne	Cat#DY270
Human TNF-alpha DuoSet ELISA	Bio-techne	Cat#DY210
TMB Substrate Reagent Set	BD Biosciences	555214; CAS: 67-56-1

**Deposited data**		

Full Western blot images	Mendeley	https://doi.org/10.17632/hb5t3r68yh.1

**Experimental models: Cell lines**		

293T	Resource Center-Cellulonet, Lyon, France	ATCC # CRL-3216
U937	Resource Center-Cellulonet, Lyon, France	ATCC # CRL-1593.2

**Oligonucleotides**		

Primers for sgRNA, see Table S2	This paper	N/A
Primers for Sequencing, see Table S2	This paper	N/A
Primers for mutagenesis, see Table S2	This paper	N/A
Primers for qRT-PCR, see Table S2	This paper	N/A

**Recombinant DNA**		

pSIRV-NF-kB-eGFP	Addgene	Cat#118093
pENTR1A	ThermoFisher Scientific	Cat#A10462
pInducer20	Meerbrey et al.^46^	pInducer20
pKLV-U6gRNA(BbsI)-PGKpuro2ABFP	Addgene	Cat#50946
pMD2.G	Addgene	Cat#12259
psPAX2	Addgene	Cat#12260
pKLV-BFP-sgALPK1ex2	This paper	N/A
pKLV-BFP-sgALPK1ex9	This paper	N/A
pNF-kB-Luc	Stratagene	Cat#219077
pRL-TK	Promega	Cat#E2231
pKLV-BFP-sgTIFAex2#1	This paper	N/A
pKLV-BFP-sgTIFAex2#1	This paper	N/A
p20-ALPK1	This paper	N/A
p20-TIFA	This paper	N/A
p20-ALPK1p.D76H	This paper	N/A
p20-ALPK1p.T237M	This paper	N/A
p20-ALPK1p.Y254C	This paper	N/A
p20-ALPK1p.D342H	This paper	N/A
p20-ALPK1p.A492S	This paper	N/A
p20-ALPK1p.S924P	This paper	N/A
p20-ALPK1p.A1010T	This paper	N/A
p20-ALPK1p.V1092A	This paper	N/A
Human sgRNA library Brunello in lentiGuide-Puro	Addgene	Cat#73178

**Software and algorithms**		

Compass Simple Western software	Bio-Techne	version 6.1.0
sequence trace decomposition software TIDE	Brinkman et al.^45^	https://tide.nki.nl
Inkscape	1.2.2	https://inkscape.org
FlowJo	Becton Dickinson version 10.10.0	https://www.flowjo.com
Graph Pad 10.1	Prism version 10.1	https://www.graphpad.com
ChiP-Atlas	Zou et al.^49^	https://chip-atlas.org
Integrative Genomics Viewer software	IGV_2.16.2	https://igv.org

### Experimental models and study participant details

#### Ethics

The study was approved by the French Comité de Protection des Personnes Ile de France 1 (CPP,# 2022-A00682-41). The authors observed a strict accordance to the Helsinki Declaration guidelines. Healthy donor (HD) blood was provided by the Etablissement Français du Sang (EFS) in the framework of the convention # EFS AURA 21–168. Informed consent was received from participants prior to inclusion in the study.

#### Subjects

Four patients with ROSAH syndrome from three unrelated families all bearing the p.T237M mutation were included from two clinical centers (Patient details including age and sex can be found in Table S1). The patients were not in flares at the time of inclusion. Blood samples from HD were drawn on the same day as patients. HD were anonymous and EFS did not provide information on sex and/or gender. The analysis on the influence (or association) of sex, gender, ancestry, race and ethnicity, socioeconomic status, or a combination of these factors on the results of the present study could not be provided which may limit the generalization of the results of the present study. The number of patients/HD in each experiment is presented in the figure legends.

#### Cell lines

The human myeloid cell line U937 (Obtained from Cellulonet, Lyon, France, ATCC reference CRL-1593.2) was grown in complete DMEM medium (DMEM supplemented with 10% fetal calf serum). The U937 cell line was derived by Sundstrom and Nilsson in 1974 from malignant cells obtained from the pleural effusion of a white male 37 years old patient with histiocytic lymphoma (source ATCC).

293T cells (Obtained from Cellulonet, Lyon, France, ATCC reference CRL-3216) were grown in complete DMEM medium (DMEM supplemented with 10% fetal calf serum). The 293T cell line, originally referred to as 293tsA1609*neo*, is a highly transfectable derivative of human embryonic kidney 293 cells, and contains the SV40 T-antigen. The cell are derived from the kidney of a female fetus (source ATCC).

None of the cell lines has been authenticated. All parental cell lines were tested mycoplasma-free in 2023.

### Method details

#### Experimental design

All experiments involving cell activation were performed at 37°C. All experiments have been replicated. The number of replicates, independent experiments, and HD/patients included are presented in the figure legends. No data points were excluded. Statistical methods are presented below.

#### Cell isolation

Blood was drawn in heparin-coated tubes. Peripheral blood mononuclear cells (PBMCs) were isolated by density-gradient centrifugation using Lymphocyte Separation Medium (Eurobio).^41^ Monocytes and the CD14^−^ cell fraction were isolated from PBMCs by magnetic selection using CD14 MicroBeads (Miltenyi Biotec)^42^ and the AutoMACS Pro Separator (Miltenyi Biotec) following manufacturer’s instructions. B cells were isolated from CD14^−^ cell fraction using CD19 Microbeads (Miltenyi Biotec). Cells were enumerated in the presence of a viability marker (propidium iodide, 10 μg/mL) by flow cytometry (BD Accuri C6 Flow Cytometer).^43^

#### Immunostaining and flow cytometry

PBMCs were primed or not with IFN-γ (Immunotools, 1,000 u/mL) for 16 h in complete medium (RPMI 1640, GlutaMAX medium (Thermofisher) supplemented with 10% fetal calf serum (Lonza). For phosphoFlow experiments, cells were treated as indicated with ADP-heptose (Invivogen, 10 μM), PMA (Invivogen, tlrl-pma, 50 nM), ultra-pure LPS (Invivogen, 100 ng/mL) for 30 min in a 37°C water bath in 200 μL. At the end of the stimulation, cells were rapidly fixed by addition of 2 mL of fixative buffer (BD Phosflow Lyse/fix buffer, #55049) and further incubating the cells at 37°C for 10 min. Cells were then pelleted by centrifugation (500 g, 2 min at 4°C), transferred in microplate, washed once in ice-cold FACS buffer (PBS, 2% FCS, 1mM EDTA) before permeabilization (BD Perm buffer II, #558052, 100 μL per well during 30 min on ice). Cells were washed once and incubated in blocking buffer (10% FCS in BD Perm/Wash buffer #554723) for 10 min. After an additional wash, cells were stained for 30 min at 4°C with the relevant antibodies (key resources table) diluted in Perm/Wash buffer. Cells were washed twice and analyzed on a BD LSR Fortessa analyser. For IL-8 intracellular staining, cells were treated with ADP-heptose in the presence of BD GolgiStop (BD Bioscience, #51-2092K2) for 12 h. Cells were treated with BD Cytofix/Cytoperm buffer (BD Bioscience, #51-2090K2) for 20 min at 4°C, staining was performed with the relevant antibodies (Key Supplemental Table) diluted in BD Perm/Wash buffer (BD Bioscience, #554723) during 30 min at 4°C. For CD69 staining, CD14^+^ cells or CD14^−^ cells separated using magnetic selection as explained above were treated with ADP-heptose (Invivogen, 10 μM) for 6 h at 37°C in complete medium. At the end of the stimulation, cells were washed once in FACS buffer, stained for 30 min at 4°C with the relevant antibodies (Key Supplemental Table) diluted in FACS buffer. Cells were washed twice and immediately analyzed on a BD LSR Fortessa analyser.

#### Cell activation

Primary monocytes were seeded in 96-well plates at 1 × 10^4^ cells/well, in complete medium. Monocytes were incubated for 3–6 h as indicated in the presence of ADP-heptose (1 μM, Invivogen).

U937 cells were seeded in 96-well plates at 5 × 10^4^ cells/well, in complete medium, primed with IFN-γ (Immunotools, 1,000 u/mL) or when applicable with IFN-α2 (Biovision #4595-100; 1,000 u/mL) or IFN-β1 (Immunotools #11343520, 1,000 u/mL) for 16 h and stimulated with ADP-heptose as indicated for 3–6 h.

Whenever applicable cells were treated with IL-6 (100 ng/mL, Immunotools), IL-8 (100 ng/mL, Immunotools), TNF (100 ng/mL, Immunotools) for 16 h or with the ALPK1 inhibitor (ALPK1-IN-2, MedChemExpress, HY147562) at 10 μM 30 min before ALPK1 addition, or with Ruxolitinib (Euromedex, #SE-S1378), Tofacitinib (MedChemExpress-#HY-40354), Upadacitinib (MedChemExpress-#HY-19569), Deucravacitinib (MedChemExpress-#HY-117287) at 1 μM or at the indicated concentrations 30 min before IFN-γ addition.

#### Genetic manipulation

Plasmids used in this study are presented in Key Supplemental Table. pSIRV-NF-kB-eGFP was a gift from Peter Steinberger (Addgene plasmid # 118093; http://n2t.net/addgene:118093; RRID:Addgene_118093). *ALPK1*^KO^, and *TIFA*^KO^ cell lines were generated by CRISPR/Cas9-mediated gene invalidation as previously described.^44^ The sgRNA targeting *ALPK1* and *TIFA* (Table S2) were selected from the Brunello library (Addgene) and cloned into the pKLV-U6gRNA(BbsI)-PGKpuro2ABFP vector (from Kosuke Yusa; Addgene plasmid # 50946). sgRNA plasmids were transduced in a previously described Cas9-expressing U937 clone^44^ by spinoculation. The resulting cell lines were selected with 2 μg/mL Puromycin (Invivogen) at 72 h post-transduction for 2 weeks. The knockout cells were kept polyclonal to avoid single cell clones response bias and were screened by Western blotting analysis or sequencing of a PCR fragment corresponding to the genomic region flanking the targeted sequence. The obtained sequence files were analyzed using the sequence trace decomposition software TIDE.^45^

*TIFA*, *ALPK1 WT* and genetic variants were cloned into the pINDUCER20 plasmid^46^ under the control of a doxycycline-inducible promoter through the pENTR1A (Invitrogen) vector using a synthetic DNA fragment (TWIST Bioscience) encoding the WT proteins. The point mutants were generated using the QuickChange II site-directed mutagenesis kit (Agilent) and primers indicated in Table S2. Lentiviral particles were produced in 293T cells using pMD2.G and psPAX2 (from Didier Trono, Addgene plasmids #12259 and #12260), and pINDUCER-20 plasmids expressing the relevant transgene or the pSIRV-NF-kB-eGFP plasmid.

The various U937 cell lines were transduced by spinoculation. U937 cells expressing NF-kB-eGFP were sorted at day 7 post-transduction based on GFP expression on an Aria cell sorter. A single cell clone was selected based on low GFP background and high inducibility in response to IFN-γ (1,000 u/mL) and ADP-heptose (50 nM). U937 cells expressing TIFA and ALPK1 constructs were selected during 10 days with Geneticin (Sigma, #A1720) at 500 μg/mL. TIFA and ALPK1 expression were induced by treatment with doxycycline (1 μg/mL) for 16 h before stimulation. All parental cell lines were tested for mycoplasma contamination.

#### Immunoblot analysis

Cells were lysed in 25 mM TrisHCl, 150 mM NaCl, 1 mM EDTA and 0.1% NP-40 buffer containing Mini Protease Inhibitor Mixture (Roche) and sodium fluoride (Sigma) by a quick freezing and thawing step. Proteins were separated by SDS/PAGE on precast 4–15% acrylamide gels (Bio-rad) and transferred to TransBlot Turbo Midi-size PVDF membranes (Bio-rad). Alternatively, the Jess Simple Western system (ProteinSimple, San Jose CA, USA), a size-based automated capillary Western blot assay, was used. The manufacturer’s standard method for 12-230-kDa Jess separation module (SM-W004) was used. Digital images of the capillary chemiluminescence were captured using Compass Simple Western software (version 6.1.0, Protein Simple) which automatically computed the apparent molecular weight.

Antibodies used are presented in Key Supplemental Table. Cell lysates were reprobed with a mouse monoclonal antibody anti-β-actin (clone C4, Millipore; 1:5,000 dilution). Full Western blot images are available on Mendeley https://doi.org/10.17632/hb5t3r68yh.1.

#### Cytokine detection

Levels of IL-8, TNF and CCL3 in monocyte supernatants were quantified by ELISA (R&D Systems #DY208, DY210, and DY270, respectively).

#### Transcript level analyses

Transcript levels at steady state in blood cells (classical monocytes, naive CD4^+^ T cells and naive B cells, TCM and TEM) as determined by RNAseq were extracted from the DICE database^25^ or from Protein atlas.^47^ Transcript levels in primary human macrophages as determined by RNAseq were extracted from the GSE82227 dataset.^48^ For real-time PCR, total RNA was extracted with TRI Reagent (Sigma-Aldrich) or Nucleospin RNA kit (Macherey Nagel, #740955) and reverse transcribed with random primers and OligodT primers combined with ImProm-II Reverse Transcription System (Promega). Quantitative real-time PCR was performed using FastStart Universal SYBR Green Master Mix (Roche) and an Applied StepOnePlus Real-Time PCR Systems (ThermoFisher Scientific). Gene-specific transcript levels were normalized to the amount of human *ACTB* transcripts. Primers sequences are indicated in the Table S2.

#### ChipSeq analyses

STAT1 ChipSeq analyses were performed using ChiP-Atlas^49^ and the Integrative Genomics Viewer software (IGV_2.16.2)^50^ using the genome GRCh38/hg38 and the following public dataset (GSE 43036, human monocytes^51^).

#### NF-kB reporter assays

U937 expressing eGFP under the control of an NF-kB responsive promoter were seeded at 2.5 10^5^ cells per well in 48 well plates and treated with IFN-γ (1,000 u/mL) for 16 h followed by ADP-heptose (100 nM). 6 h later, cells were collected and analyzed for eGFP on a flow cytometer (BD LSR Fortessa). FlowJo 10.9.0 software was used to calculate the mean fluorescence intensity and to generate concatenates.

pNF-kB-Luc plasmid (Stratagene, #219077) expressing the firefly luciferase under the control of an NF-kB responsive promoter was cotransfected with the pRL-TK plasmid (Promega) encoding renilla luciferase under a constitutive promoter, and ALPK1 variants-containing pInducer20 plasmids, in 293T cells (Cellunonet, Lyon, France) using FuGENE HD Transfection Reagent (Promega, #E2311). 6 h later, cells were treated or not with doxycycline (1 μg/mL) to induce ALPK1 expression. 16 h later, cells were treated or not with ADP-heptose (1 μM). 3 h later, cells were lysed using the Dual-Glo Luciferase Reagent (Promega, #E2940) and luminescence signals were acquired on a Tristar luminometer following the manufacturer instructions. Firefly luciferase signals were normalized to renilla luciferase signals.

### Quantification and statistical analysis

All tests were two sided at the 0.05 significance level. Statistical analyses were carried out with Graph Pad Prism 10.1 and each test and statistical parameters are presented in each figure legends. Normality was performed using Shapiro-Wilk test. Paired measures with normal distribution were analyzed by RM one-way ANOVA, with the Geisser-Greenhouse correction (equal variability of differences not assumed) and Holm-Šidák’s multiple comparisons test, with individual variances computed for each comparison. Paired measures that did not verified the normal distribution were analyzed by Friedman test with Dunn’s multiple comparisons test. Unpaired values that did not verified the normal distribution were compared using Kruskal-Wallis test with Dunn/s multiple comparison test. Unpaired values that verified the normal distribution were compared using One way ANOVA test with Šidáks’ correction for multiple tests. Single comparisons of paired values that verified the normal distribution were performed using paired t-tests. Adjusted *p*-values are shown with the following nomenclature: n.s.: *p* > 0.05; ∗: 0.01 < *p* < 0.05; ∗∗: 0.001 < *p* < 0.01; ∗∗∗: *p* < 0.001.
