# Supplementary material for: IFN-γ licenses normal and pathogenic ALPK1/TIFA pathway in human monocytes
Source: iScience. 2024 Dec 10;28(1):111563. doi: 10.1016/j.isci.2024.111563 (PMC11758396; doi:10.1016/j.isci.2024.111563)
Supplement: Document S1. Figures S1–S5, Tables S1, and S2 [file mmc1.pdf]

iScience, Volume *28*

Supplemental information

IFN-γ licenses normal and pathogenic ALPK1/TIFA pathway in human monocytes

Amandine Martin, Solène Caron, Mélissa Marcotte, Pauline Bronnec, Etienne Garneret, Nora Martel, Georgina Maalouf, Pascal Sève, David Saadoun, Yvan Jamilloux, and Thomas Henry

Patient	*ALPK1 mutation*	*Age*	*Sex*	*Treatments*	*Flare at sampling*
RP1	p.T237M	31	M	Cortancyl 20 mg/day, Imurel 50 mg/ 3X day, Infliximab 120 mg/ 2x a month	No
RP2	p.T237M	54	F	None	No
RP3	p.T237M	31	M	Cortancyl 5mg/day	No
RP4	p.T237M	44	M	None	No

Supplemental Table S1 (related to STAR Methods): ROSAH Patients included in this study.

Name	*Sequence (5'-3')*	*Usage*
sgRNA TIFA-1 for	CACCCATCCTGGCCAGTTGCAGTGGT	KO of TIFA
sgRNA TIFA-1 rev	TAAAACCACTGCAACTGGCCAGGATG	KO of TIFA
sgRNA TIFA-2 for	CACCGAAACTCCCTTCCAGCGAAGGT	KO of TIFA
sgRNA TIFA-2 rev	TAAAACCTTCGCTGGAAGGGAGTTTC	KO of TIFA
sgRNA ALPK1-ex2 for	CACCCATCCTCGCTCGGGACTGTGGT	KO of ALPK1
sgRNA ALPK1-ex2 rev	TAAAACCACAGTCCCGAGCGAGGATG	KO of ALPK1
sgRNA ALPK1-ex9 for	CACCTGCCAGTATACCTAGCGACGGT	KO of ALPK1
sgRNA ALPK1-ex9 rev	TAAAACCGTCGCTAGGTATACTGGCA	KO of ALPK1
ALPK1ex2 for	ACCCTATGCGCTTTAGTATTGTT	Sequencing of ALPK1 exon 2
ALPK1ex2 rev	TCAAGTAGCTCGGGAGGGAA	Sequencing of ALPK1 exon 2
ALPK1ex9 for	CCCATGTGCCCTAGGATGTG	Sequencing of ALPK1 exon 9
ALPK1ex9 rev	CTGGAACAAGGGACTGATGCT	Sequencing of ALPK1 exon 9
TIFA for	AGATAGGAGAAGCTAGTGTAGCA	Sequencing of TIFA exon 2
TIFA rev	ATACTCCGGTATGGGCCTGT	Sequencing of TIFA exon 2
D76H FW	CGGGCCCGAACATAAGACG	U937 cALPK1 D76H cell line
D76H RV	CGTCTTATGTTCGGGCCCG	U937 cALPK1 D76H cell line
D342H FW	GAAACGGGACCACGAACCC	U937 cALPK1 D342H cell line
D342H RV	GGGTTCGTGGTCCCGTTTC	U937 cALPK1 D342H cell line
A492S FW	GTTTGTATAACCTCACTGAAGAC	U937 cALPK1 A492S cell line
A492S RV	GTCTTCAGTGAGGTTATACAAAC	U937 cALPK1 A492S cell line
S924P FW	GTAGTCAAAATCCTTCCTCTAGC	U937 cALPK1 S924P cell line
S924P RV	GCTAGAGGAAGGATTTTGACTAC	U937 cALPK1 S924P cell line
A1010T FW	CTGCATCGCACTCACAGCG	U937 cALPK1 A1010T cell line
A1010T RV	CGCTGTGAGTGCGATGCAG	U937 cALPK1 A1010T cell line
V1092A FW	CCCAACATTACGCCACTGAG	U937 cALPK1 V1092A cell line
V1092A RV	CTCAGTGGCGTAATGTTGGG	U937 cALPK1 V1092A cell line
Y254C FW	GAATGACTGCGAGAAATTCAAGAATAACC	U937 cALPK1 Y254C cell line
Y254C RV	GGTTATTCTTGAATTTCTCGCAGTCATTC	U937 cALPK1 Y254C cell line
ALPK1 for	TGACCACCATTTGCTGTCC	qRT-PCR ALPK1 [52]
ALPK1 rev	ACGTGCCACGGATATTCAC	qRT-PCR ALPK1
TIFA for	TGGTAAACCGTCATCTGGAG	qRT-PCR TIFA [53]
TIFA rev	GAGTTCACTGACTCCCCAGC	qRT-PCR TIFA
TRAF6 for	GCGCACTAGAACGAGCAAG	qRT-PCR TRAF6
TRAF6 rev	TGGCAGTTCCACCCACACTA	qRT-PCR TRAF6
Beta actin for	ATTGGCAATGAGCGGTTC	qRT-PCR beta Actin
Beta actin rev	CGTGGATGCCACAGGACT	qRT-PCR beta Actin
ALPK1 Transgene For	CCTTCGGGCGTCAATACTTG	qRT-PCR ALPK1 transgene
ALPK1 Transgene Rev	TGATGACGACTTGTGGAGCT	qRT-PCR ALPK1 transgene
TIFA Transgene For	CGCTGTATGGTGAGGTTTGG	qRT-PCR TIFA transgene
TIFA Transgene Rev	CTCGGTGGGTGAAGAAGACT	qRT-PCR TIFA transgene
IL1-F(2)	GCACGATGCACCTGTACGAT	qRT-PCR IL1 beta
IL1-R(2)	CACCAAGCTTTTTTGCTGTGAGT	qRT-PCR IL1 beta

Table S2 (related to STAR Methods): oligonucleotides used in this study

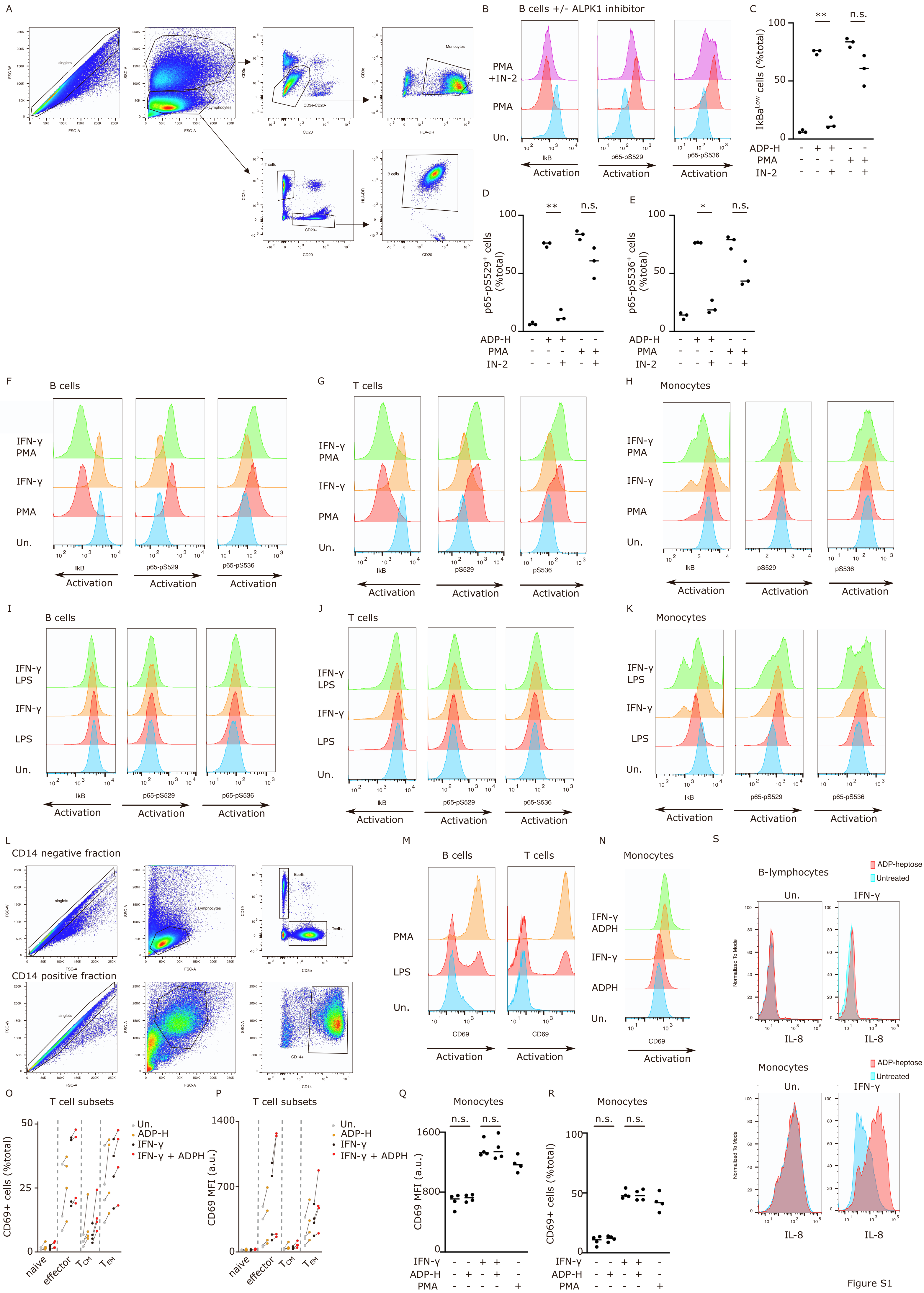

B cells +/- ALPK1 inhibitor
PMA +IN-2
PMA
Un.
IkB
p65-pS529
p65-pS536
Activation
IkBa^Low cells (%total)
ADP-H
PMA
IN-2
p65-pS529+ cells (%total)
p65-pS536+ cells (%total)
B cells
T cells
Monocytes
IFN-γ PMA
IFN-γ
IFN-γ LPS
LPS
CD14 negative fraction
CD14 positive fraction
CD69
ADPH
IFN-γ ADPH
B-lymphocytes
ADP-heptose
Untreated
IL-8
T cell subsets
CD69+ cells (%total)
CD69 MFI (a.u.)
Un.
ADP-H
IFN-γ + ADPH
naive
effector
TCM
TEM

Figure S1

Supplemental Fig. S1 (related to Fig 1): Gating strategy, NF-κB activation, IL-8 and CD69 up-regulation in response to ADP-Heptose, PMA and LPS in T cell subsets, B cells and monocytes.

(A) PBMCs gating strategy in the phosphoFlow experiments to define monocytes, T and B cells. (B-E) $CD14^-$ cells or (F-K) PBMCs from 3 healthy donors were primed or not with IFN-γ for 16 h followed by 30 minutes stimulation with (B-E) ADP-heptose (ADP-H) at 10 μM. (B-H) Phorbol Myristate Acetate (PMA) at 100 ng/mL or (I-K) lipopolysaccharide (LPS) at 100 ng/mL. (B-E) When applicable, cells were pretreated with ALPK1 inhibitor (IN-2) at 10 μM 30 minutes before addition of ADP-H or PMA. IκBα (left panel), NF-κB p65 phospho-serine 529 (middle panel) and, NF-κB p65 phospho-serine 536 (right panel) were detected by flow cytometry following gating on (B-F, I) $CD3\varepsilon^-$ $HLA\text{-}DR^+CD20^+$ cells (B cells), (G, J) $CD20^-CD3\varepsilon^+$ cells (T cells) and (H-K) $CD19^-$, $CD3\varepsilon^-$, $HLA\text{-}DR^+$ cells (monocytes). (C-E) the percentage of $I\kappa B\alpha^{low}$ (C), $p65\text{-}pS529^+$ (D), $p65\text{-}pS536^+$ (E) among total B cells is shown. (L) Gating strategy on the $CD14^+$ and $CD14^-$ fractions collected from PBMCs post-magnetic beads separation. (M) $CD14^-$ cells from 4 healthy donors were primed or not with IFN-γ for 16 h followed by 6 h stimulation with LPS at 100 ng/mL. CD69 was detected by flow cytometry following gating on $CD3\varepsilon^-CD19^+$ cells (B cells, left panel) and, $CD19^-CD3\varepsilon^+$ cells (T cells, right panel). (O-P) $CD14^-$ cells from 4 healthy donors were primed or not with IFN-γ for 16 h followed by 6 h stimulation with ADP-heptose (ADP-H) at 1 μM. The percentage of $CD69^+$ cells (O) and CD69 mean fluorescence intensity (MFI) (P, a.u., arbitrary units) is shown in naïve ($CD19^-$, $CD3e^+$, $CD45RA^+$, $CD197^+$), effector ($CD19^-$, $CD3e^+$, $CD45RA^+$, $CD197^-$), central memory (TCM: $CD19^-$, $CD3e^+$, $CD45RA^-$, $CD197^+$) and effector memory (TEM: $CD19^-$, $CD3e^+$, $CD45RA^-$, $CD197^-$) cells. (Q) CD69 MFI and (R) the percentage of $CD69^+$ cells in total monocytes is shown. (S) PBMCs from 3 healthy donors were treated or not with IFN-γ and treated (red histograms) or not (blue) with ADP-Heptose at 10 μM for 12 h in the presence of Golgi-block. IL-8 intracellular staining in $CD20^+$, $CD3\varepsilon^-$, $HLA\text{-}DR^+$ cells (B lymphocytes-top panels), and $CD20^-$, $CD3\varepsilon^-$, $HLA\text{-}DR^+$ cells (monocytes-bottom panels) is shown. (C-E, Q-R) One dot corresponds to the value from one healthy donor, the bar corresponds to the median of 3 (C-E) to 4 (Q-R) healthy donors. (O-P) One dot corresponds to the value from one healthy donor, each line links the values obtained for each donor in the presence or absence of ADP-Heptose. (A-B, F-N, S) Concatenates from three (A-B, F-K, S) to four (L-N) healthy donors (one experiment representative of three independent experiments) are shown. (Q): Friedman paired test with Dunn's correction for multiple tests was performed (n.s.: $p>0.99$). (C-E, R) One-way ANOVA with Šidáks' correction for multiple tests was performed ((C): **: p=0.007, n.s.: p=0.169; (D): **: p=0.007, n.s.: p=0.182; (E): *: p=0.0118, n.s.: p=0.084; (R): n.s.: p=0.73, p=0.99, from left to right, respectively).

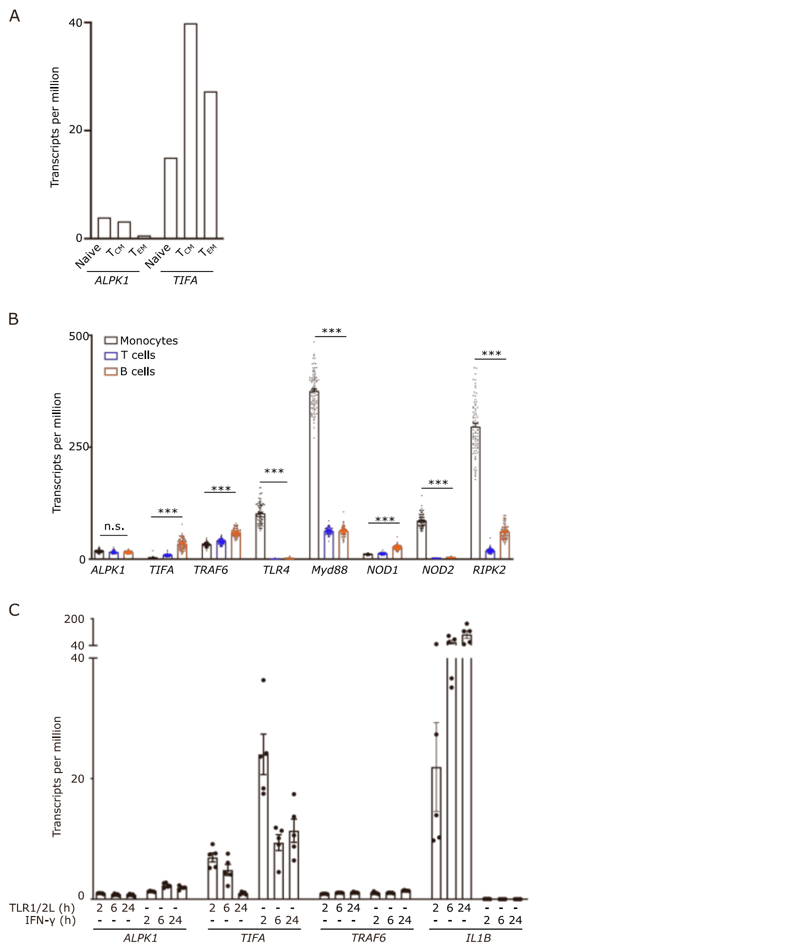

<u>Supplemental Fig. S2 (related to Fig. 2): Expression of innate immune receptors and adaptors at steady state in monocytes, T and B cells and regulation of *ALPK1/TIFA/TRAF6/IL1B* transcript levels in macrophages.</u>

(A) *ALPK1* and *TIFA* transcript levels in naïve, central memory and effector memory CD8 T cells were obtained from the protein Atlas database. (B) *ALPK1*, *TIFA, TRAF6, TLR4, MYD88, NOD1, NOD2, RIPK2* transcript levels in monocytes (black), naïve T cells (blue) or naïve B cells (orange) were obtained from the public RNAseq database DICE. Kruskal-Wallis unpaired test with Dunn's correction for multiple tests was performed (n.s.: p>0.99; ***: p<0.001). Each dot represents the value from one healthy donor, the bar represents the mean +/- SEM. (C) Primary monocytes-derived macrophages from 5 healthy donors were treated with a TLR1/2 ligand (19kDa triacylated lipopeptide from *Mycobacteria*) or IFN-γ for the indicated time. *ALPK1*, *TIFA, TRAF6* and *IL1B* transcript levels were analyzed by RNAseq (GSE82227 dataset [48]) and normalized to the untreated sample. Each dot represents the value from one individual, the bar represents the mean +/- SEM.

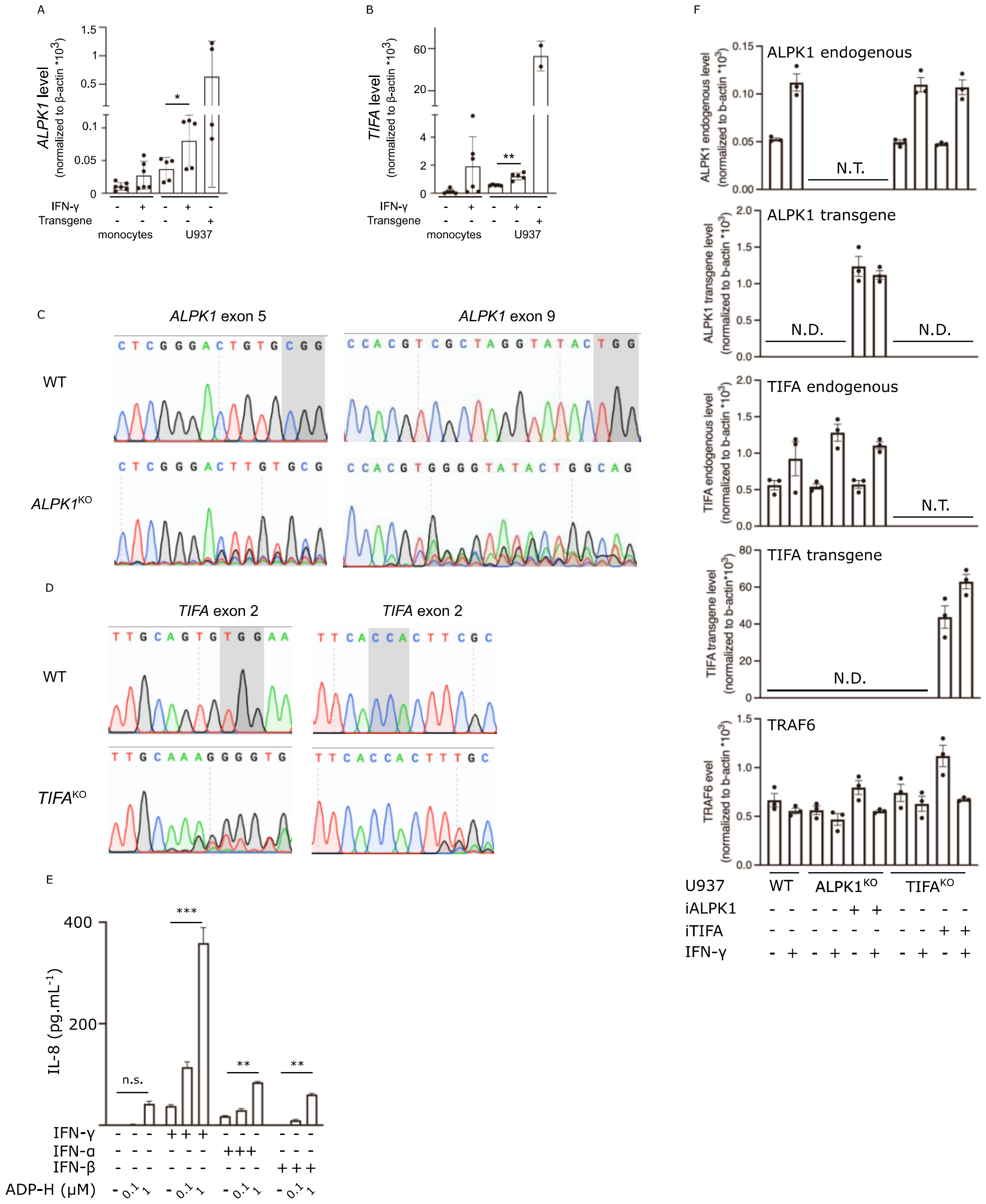

Supplemental Fig. S3 (related to Fig. 3): *ALPK1* and *TIFA* are efficiently invalidated by CRISPR and type I IFNs potentiate IL-8 production in monocytes although not as strongly as IFN-γ.

ALPK1 (A) and *TIFA* (B) transcript levels were quantified in primary human monocytes and U937 cells in the presence or not of IFN-γ treatment (16 h). U937 expressing the transgene iALPK1(A) or iTIFA (B) are shown for comparison. Each dot represents the mean value of triplicate values from one healthy donor or one U937 cell line. The bar represents the mean +/- SD. T-tests were performed *: p=0.011; **: p=0.004. Sequencing of *ALPK1* (C) and *TIFA* (D) in U937 WT, *ALPK1*^{KO} and *TIFA*^{KO} cells. The position of the PAM sequence is indicated in grey. Two sgRNA per cell line were used. (C) Tracking of indels by decomposition (TIDE) determined that >91% and >61% of sequences were knocked-out at the exon 5 and 9 loci of *ALPK1*, respectively. (D) >80% of sequences differed from the WT sequence at the two exon 2

loci. Due to the presence of deletions, TIDE could not be used. (E) U937 were primed or not with IFN-γ, IFN-α2 or IFN-β and treated with ADP-heptose at the indicated concentrations. IL-8 concentrations were quantified in the supernatant at 3h post-treatment. Each dot represents one biological replicate, the bar represents the mean +/- SEM from three replicates. One experiment is shown. One-Way Anova with Šidák's correction for multiple tests was performed ns: $p=0.06$, ***$p<0.001$; **: $p=0.002$ and $p=0.006$ from left to right, respectively. (F) qRT-PCR were performed in the indicated doxycycline-treated U937 cell lines in the presence or not of IFN-γ. Results from qRT-PCR specific for endogenous or transgene *ALPK1* and *TIFA* are shown.

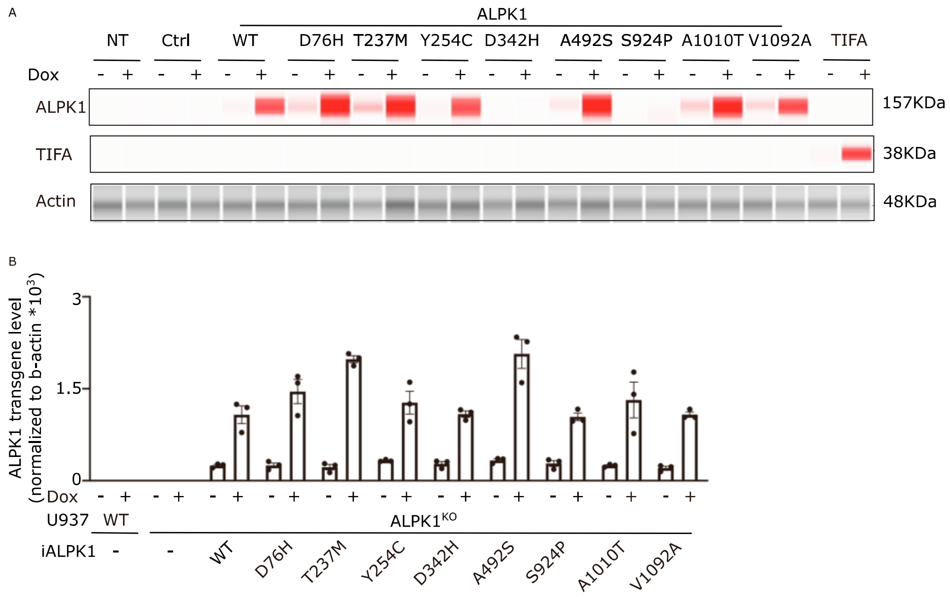

Supplemental Fig. S4 (Related to Fig. 4): Expression of *ALPK1* and *TIFA* transgenes.

(A) 293T were transfected with the plasmid encoding the indicated doxycycline-inducible ALPK1 variants, PSTPIP1 as a control (Ctrl) or TIFA. Cells were exposed or not to doxycycline for 16 h before analysis of ALPK1, TIFA and β-actin protein levels by capillary-based western blot analysis. The computed molecular weight is indicated. (B) U937 cell lines expressing the indicated doxycycline-inducible ALPK1 variants were treated as indicated with doxycycline for 16 h before analysis of *ALPK1* transgene and *β-actin* levels by qRT-PCR analysis. Each dot represents one sample, the bar represents the mean +/- SEM of one experiment.

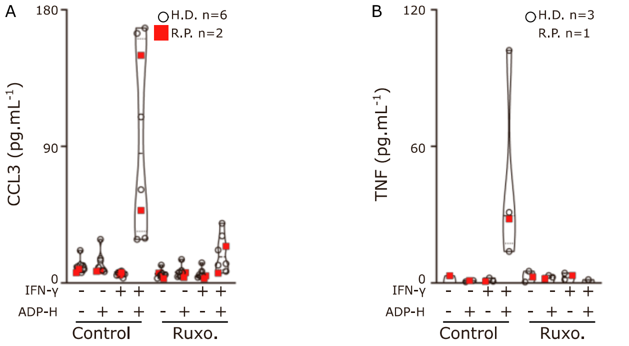

Supplemental Fig. S5 (Related to Fig. 5): JAK inhibitors block IFN-γ-mediated ALPK1 responses

(A-B) Primary monocytes from HD (open circle, (A) n=6, (B) n=3) or ROSAH syndrome patients (red square, (A) n=2, (B) n=1) were primed or not with IFN-γ (1,000 u/mL) for 16 h in the presence of ruxolitinib (1 µM) as indicated, and then treated by ADP-heptose (1 µM) for 6 h. (A) CCL3 and (B) TNF levels were quantified in the supernatant by ELISA. Each symbol represents the average value from a biological triplicate from one individual, the line shows the median and the dotted lines the quartile.

Supplemental References

52. Liao H.-F., Lee H.-H., Chang Y.-S., Lin C.-L., Liu T.-Y., Chen Y.-C., Yen J.-C., Lee Y.-T., Lin C.-Y., Wu S.-H., et al. Down-regulated and Commonly mutated ALPK1 in Lung and Colorectal Cancers. Sci. Rep. 2016;6:27350. doi:10.1038/srep27350.

53. Zimmermann S., Pfannkuch L., Al-Zeer M.A., Bartfeld S., Koch M., Liu J., Rechner C., Soerensen M., Sokolova O., Zamyatina A., et al. ALPK1- and TIFA-Dependent Innate Immune Response Triggered by the Helicobacter pylori Type IV Secretion System. Cell Rep. 2017;20:2384–2395. doi:10.1016/j.celrep.2017.08.039.
